# Chitosan and Hydroxyapatite Based Biomaterials to Circumvent Periprosthetic Joint Infections

**DOI:** 10.3390/ma14040804

**Published:** 2021-02-08

**Authors:** Ana Rita Costa-Pinto, Ana Luísa Lemos, Freni Kekhasharú Tavaria, Manuela Pintado

**Affiliations:** Universidade Católica Portuguesa, CBQF-Centro de Biotecnologia e Química Fina-Laboratório Associado, Escola Superior de Biotecnologia, Rua Diogo Botelho 1327, 4169-005 Porto, Portugal; aluisa_lemos@hotmail.com (A.L.L.); ftavaria@porto.ucp.pt (F.K.T.)

**Keywords:** biomaterials, chitosan, hydroxyapatite, antimicrobial, periprosthetic joint infection, osteoregeneration

## Abstract

Every year, worldwide, millions of people suffering from joint pain undergo joint replacement. For most patients, joint arthroplasty reduces pain and improve function, though a small fraction will experience implant failure. One of the main reasons includes prosthetic joint infection (PJI), involving the prosthesis and adjacent tissues. Few microorganisms (MO) are required to inoculate the implant, resulting in the formation of a biofilm on its surface. Standard treatment includes not only removal of the infected prosthesis but also the elimination of necrotic bone fragments, local and/or systemic administration of antibiotics, and revision arthroplasty with a new prosthesis, immediately after the infection is cleared. Therefore, an alternative to the conventional therapeutics would be the incorporation of natural antimicrobial compounds into the prosthesis. Chitosan (Ch) is a potential valuable biomaterial presenting properties such as biocompatibility, biodegradability, low immunogenicity, wound healing ability, antimicrobial activity, and anti-inflammatory potential. Regarding its antimicrobial activity, Gram-negative and Gram-positive bacteria, as well as fungi are highly susceptible to chitosan. Calcium phosphate (CaP)-based materials are commonly utilized in orthopedic and dentistry for their excellent biocompatibility and bioactivity, particularly in the establishment of cohesive bone bonding that yields effective and rapid osteointegration. At present, the majority of CaP-based materials are synthetic, which conducts to the depletion of the natural resources of phosphorous in the future due to the extensive use of phosphate. CaP in the form of hydroxyapatite (HAp) may be extracted from natural sources as fish bones or scales, which are by-products of the fish food industry. Thus, this review aims to enlighten the fundamental characteristics of Ch and HAp biomaterials which makes them attractive to PJI prevention and bone regeneration, summarizing relevant studies with these biomaterials to the field.

## 1. Introduction

Implantation of medical devices has been growing in parallel with the constant development of medical care and it is expected that, at least once in their lifetime, all individuals will undertake some type of medical device implantation [1,2]. These devices can diverge from simple catheters, contact lenses, stents, or orthopedics prosthetics, for example [2]. Although biomedical implants have transformed medicine today, they also increase the risk for infection. The implantation of any surgical medical device is invasive and triggers an immune reaction due to the presence of the foreign body, creating a locus *minoris resistentiae*. This condition induces vulnerability to bacterial attack mainly by opportunistic pathogens.

In the orthopedic field, joint replacement (arthroplasty) is a life-enhancing procedure for millions of people worldwide each year [3]. This procedure is only considered as a treatment when severe joint pain or dysfunction is not alleviated by less-invasive therapies. It is indicated for different joint problems, including osteoarthritis, rheumatoid arthritis, abnormal formation or alignment of the joint, and traumatic injury derived arthritis [4,5]. Successful artificial joint replacement provides pain relief, restores function and independence to the patient. However, a minority (0.8–1.9% of knee arthroplasties and 0.3–1.7% of hip arthroplasties) will undergo device failure, requiring additional surgery [3]. The major causes of failure include loosening of the prosthesis from bone and infection at the site of implantation [6]. Orthopedic implants remain in the body, and for that reason, the infection is quite endangering, especially the formation of biofilms at their surface, which turns these types of infection particularly threatening [6]. The outcome of a revision surgery will be extremely affected by the cause of prosthesis failure because the type of treatment differs profoundly between aseptic loosening, mechanical failure, or prosthetic joint infections (PJI). For this reason, there is an increasing trend for publications about prosthetic infection and the development of antimicrobial strategies, which was the basis for the literature survey adopted in this review. Therefore, this review aims at describing the pathology of PJI, to understand the main players and to consider chitosan and hydroxyapatite as biomaterials with potential to improve this impacting health problem.

## 2. Periprosthetic Joint Infections

As Sculco [7] wrote more than 30 years ago, “Infection in total joint replacement is a devastating and life-threatening complication for the patient. It can also be an economic disaster for hospitals that treat large numbers of these patients”. While the majority of joint arthroplasties provide pain-free function, 1.63% of the patients within 2 years will experience device failure [8] and require additional surgery during the life of the device [6]. One of the reasons for implant failure is PJI, involving the prosthetic material and the adjacent tissues, being one of the most serious complications after orthopedic surgery and a foremost cause for total joint revision surgery [9,10]. The economic burden associated with a joint revision surgery is superior to primary replacement, due to its complexity and prolonged hospitalization times, with worse prognosis and higher risk of failure [11,12].

Periprosthetic joint infection is one of the most serious complications after major orthopedic surgery [8], resulting in surgical failure requiring revision arthroplasty [13] and, in severe cases, can lead to amputation and ultimately to death [14]. The absolute number of PJI cases is definitely on the rise due to the exponential increase of primary arthroplasties [15]. In the United States each year, approximately 1 million hip and knee arthroplasties are performed [3]. Currently, an increasing number of these procedures corresponds to revision surgeries [16]. Both numbers of primary total and revision procedures of hip and knee arthroplasties have exponentially increased, being projected that this number will exceed 4 million between 2005 and 2030 [3]. Although the use of aseptic conditions and antibiotic prophylaxis has substantially contributed to the reduction of the infection rates after joint arthroplasty, it seems there is a worldwide tendency to increase this problem [17]. Yokoe and co-workers [18] performed a study in California, USA showing infection rates of 2.3% after total hip and 2% after total knee arthroplasties. Data collected from a study performed at Hospital de Santo António in Porto, Portugal, between 2014 and 2015, reported that in this country, the mean cost for each aseptic revision represents more than the triple of primary uneventful arthroplasty and 1.5 times the cost of revision for causes other than infection [19]. Treatment of PJI is both costly and highly complex with extreme implications to the patient’s health, and prevention is therefore, a priority. Nevertheless, the absolute number of PJI cases will indeed continue to rise due to the increasing number of primary arthroplasties [20]. Possible sources of nosocomial infection include the operating room environment, surgical equipment, medical and paramedical staff clothing, and resident bacteria from the patient’s skin microbiota [21]. Although the control of sterility has progressed, with the existence of a laminar airflow (still controversial) in the operating room [22] and antibiotic prophylaxis [23], reducing the incidence of infections associated to orthopedic implants to less than 2% [6], these strategies do not completely eradicate the risk of infection. Nevertheless, it remains a significant clinical problem, due to the huge impact in terms of mortality and morbidity, the complex revision procedures doubling the re-hospitalization rates and imposing severe demands on healthcare resources [24]. In addition to all of these complications, it is important to refer that the treatment with antibiotics contributes to antimicrobial resistance [25].

Some factors increase the patient risk to be infected, including comorbidities such as obesity, poor nutritional status, rheumatoid arthritis, diabetes mellitus, and superficial wound complications such as surgical site infection [26,27]. Some studies reported obesity as a major risk factor, probably due to the longer surgical time required and its influence over the durability of the implant prosthesis, due to the excess weight [5,28]. Rheumatoid arthritis patients, because of the treatment with immunosuppressive drugs, are also at higher risk of infection [29,30]. Diabetes is a similar condition that increases the risk of infection due to the presence of high levels of glucose, which promotes biofilm formation [31].

The classification of PJIs is not consensual in the literature, where several classifications can be found based on the etiopathogenic significance [6,14,20,32,33,34,35]. In this review, we will consider 3 types of PJIs, classified as early (less than 3 months after surgery), delayed (3–24 months), and late (more than 2 years after surgery) [33,34,35] (Table 1).

All PJIs can be acquired at the time of surgery, its aggressiveness usually arises from the virulence of the microorganisms (MO) or in the case of late infection, via bloodstream (hematogenous infection) [36].

The chosen treatment relies on the type of PJI and the degree of infection, independently of the type of the prosthesis (cemented, cementless or press-fit, and hybrid—cemented stem with uncemented cup). In the case of surgical treatment, the most used is the two-stage revision arthroplasty, which consists of two surgeries. The first one to remove all infected tissue (at least what can be perceived as necrotic and infected tissue), the cement and the prosthetic itself, followed by the implantation of a cement joint spacer impregnated with antibiotics. The patient will also be subjected to intravenous antibiotic therapy typically during 4 to 6 weeks. After that period, the patient is evaluated for any signs of infection. In the case of no infection, a second surgery to implant a new prosthesis with an anti-microbial-loaded cement is performed [37]. The one-stage arthroplasty is more controversial, mainly due to invasiveness to the patient and a high degree of failure [38]. The surgeon must be highly skilled to perform the debridement of all infected tissue and place the new prosthetic implant, in the same surgical procedure. As in the two-stage arthroplasty, the patient must perform both intravenous anti-microbial therapy [38].

### 2.1. Mechanisms of Infection

The process of bacterial adhesion to a surface (living or abiotic) and the subsequent development of a biofilm can be divided into two different attachments: first, an unspecific reversible, and second, an irreversible one [39]. The primary adhesion involves a close approximation of the microorganism with the surface, which is facilitated by the physiological fluids extracellular matrix (ECM) proteins and immune system components, that rapidly cover the implant material [40]. When the MO is close to the surface (<1 nm), the adhesion will depend on the existing forces (attractive or repulsive) [39].

Pathogenic MO usually contaminate the prosthetic implant during surgery. These MO might enter the implantation site by the surgical incision, either coming from patients’ endogenous flora or from the surgical room staff/environment. It can also arise from hematogenous spread, which might occur during the post-operative period [41]. A significant factor is the low inoculum of MO needed to establish an infection, adherence to the implant and formation of a biofilm, in which they are protected from conventional antimicrobial agents and host immune system [34]. Once the infection is settled, the infected implant must be removed and replaced, as well as potentially infected or necrotic tissues [13].

**Figure 1:**
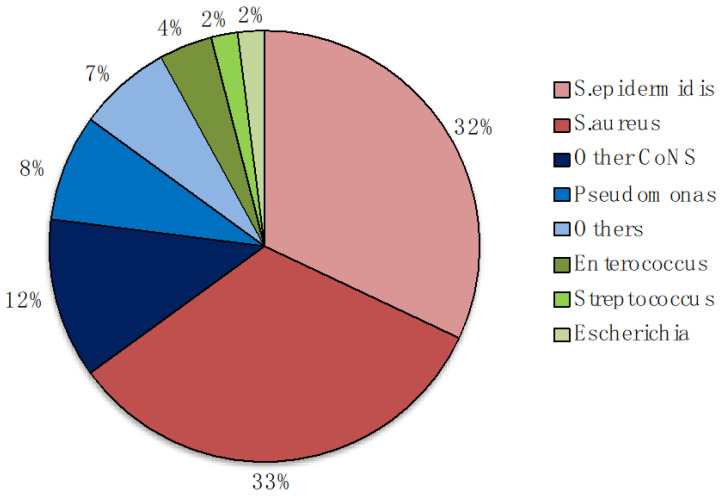
Major microorganisms (MO) associated with implants contamination in orthopedic field. Adapted from [32,42,46]. CoNS—Coagulase-negative staphylococci other than Staphylococcus epidermidis.

The large variety of strains isolated from infected implants include: (1) Gram-positive bacteria (e.g., *Enterococcus faecalis* (*E. faecalis*), *Staphylococcus aureus* (*S. aureus*) including methicillin-resistant strain (MRSA), *Staphylococcus epidermidis* (*S. epidermidis*) and other coagulase-negative Staphylococci (CoNS), *Streptococcus viridans* (*S. viridans*); and (2) Gram-negative bacteria (e.g., *Pseudomonas aeruginosa* (*P. aeruginosa*), *Escherichia coli* (*E. coli*), *Proteus mirabilis* (*P. mirabilis*)) [25,40,42]. These bacteria are the MO mainly responsible for biofilm formation on indwelling medical devices [40,43]. *S. epidermidis* and *S. aureus* are the most frequent cause with similar rate (32%) that together with *P. aeruginosa* are responsible of approximately 75% of biofilm-associated infections [44] (Figure 1). However, these MO are difficult to detect and effective treatments for their eradication are absent [45].

### 2.2. Microbial Adhesion and Biofilm Formation

Microorganisms adhesion and subsequent formation of biofilms is the leading cause of implant-associated infections, corresponding to 60% of hospital-associated infections [47,48]. Biofilm infection may cause tissue destruction, systemic dissemination of the pathogen, and malfunction of the device, resulting in serious illness and possible death [49].

**Figure 2:**
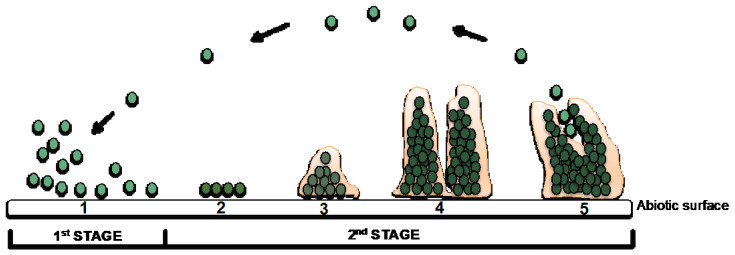
Stages of biofilm formation. First stage—contact and attachment of MO to the surface; Second stage—irreversible adhesion of MO and growth; production of a biofilm and possible colonization of other areas. 1—Aggregation (reversible); 2—adhesion (irreversible); 3—microcolony formation; 4—biofilm production; 5—dispersion.

Bacterial adhesion is influenced by several factors: (1) hydrophobicity and surface charge of bacteria; (2) surface parameters of the implant (e.g., chemical composition, charge, roughness, hydrophobicity, and wettability); (3) environmental factors (e.g., temperature, time of exposure, bacterial concentration, chemical treatment, or the presence of antibiotics); and (4) the presence of serum or tissue proteins [50,51]. Biofilms are organized and complex microbial communities enclosed in a self-produced polymer matrix, consisting of polysaccharides, proteins, and DNA, which have the capacity to adhere and grow on exposed surfaces, either on inert or living surfaces [52,53]. This process, as summarized in Figure 2, occurs accordingly to a well-known sequence of events: attachment, adhesion, aggregation, and dispersion of cells from the biofilm to initiate a new cycle of biofilm formation elsewhere.

Initially, bacteria approach and attach reversibly to the surface through non-specific physicochemical interactions, such as hydrophobic, electrostatic, and van der Waals forces [36]. Next, MO become irreversibly attached to the surface through structures like adhesins. Upon adherence, bacteria start to divide, secrete, and collect proteins, polysaccharides, and DNA to produce a biofilm. Once established, portions of the bacterial colony, called planktonic MO, might escape the biofilm and colonize other areas of the body [40,54,55]. This way of growth provides structural stability and protection from the environment due to the slow transport of molecules throughout the polysaccharide matrix [45]. Nutrient availability and metabolic interaction are improved due to the presence of water channels interspersed throughout the biofilm and contribute for the acquisition of genetic diversity [14,43,52]. These factors explain the great resistance to antibiotic treatments, as well as its tolerance to disinfectants, and resistance to phagocytosis by the immune system [36]. When the infections are not treatable using conventional techniques, the treatment includes two stages as aforementioned described: (1) the implant is removed and the infection treated, and (2) a new device is implanted [40].

Currently, several studies focusing on the use of antibiotic loaded biomaterials are being performed but most of them share limitations inherent to bacterial antibiotic resistance [54]. Perioperative antimicrobial (non-antibiotic) local carriers would be a better solution to overcome such challenges with potential to treat bone defects [56].

### 2.3. Current Strategies to Overcome Implant-Associated Infections

The approaches to prevent and control implant infections include inhibition of microbial adhesion to device surfaces through the modification of the biomaterial surface with coatings (e.g., photoactive, antibiotic-hydroxyapatite, nanostructured, nano-silver and antiseptic-based coatings or through surface absorption or material bulk impregnation with antimicrobial agents) [40,57]. Regarding orthopedic implant infections, the strategies include antibiotic prophylaxis before the surgical procedure [58] and/or antibiotic-releasing polymethylmethacrylate (PMMA) bone cements and spacer with different spectra and types of action [57]. The mostly used antibiotics are gentamicin, rifampicin, vancomycin, and tobramycin [59].

## 3. Joint Replacement

Joint replacement surgery or arthroplasty is an orthopedic procedure when non-invasive treatments like medication, physical therapy, and changes in everyday activities do not alleviate joint pain and disability caused by conditions such as osteoarthritis, rheumatoid arthritis, fracture, or others [60].

The surgery consists in removing part or all of a damaged joint (cartilage and bone) and installing hardware to allow movement without pain or limitations—an orthopedic prosthesis made of metal, plastic, ceramic, or a combination of these materials [61]. The prosthesis will simulate the shape and movement of a natural joint [62].

Traditionally, orthopedic devices are focused mostly on the mechanical properties and function of the implant, driving to its stability [63]. However, this aim may be compromised due to numerous issues, such as the patient health condition, infection, or bone healing capacity [64]. This has encouraged research to find alternative strategies to improve the biological interface implant-bone by modulating the biological milieu of the implant bed to accomplish strong bone healing.

### 3.1. Bioceramics

The manipulation of surface properties of biomaterials to control the interaction between implants and their biological surroundings have been one of the major research topics in the biomaterials field [65]. Calcium phosphate (CaP) compounds have emerged as prominent materials for biomedical applications mainly as bone substitutes due to their properties [66]. Their CaP ratio of 0.5–2.0 [66,67] makes them excellent choices for bone defects reconstruction [65]. The following table (Table 2) presents a list of CaP-based compounds and their applications.

#### 3.1.1. Hydroxyapatite Sources and Production Methods

The most widely used bioceramic is hydroxyapatite (Ca10(PO4)6(OH)2-HAp), which is the major inorganic component of bone [78,79]. The main natural sources of hydroxyapatite (HAp) are animal bones. Fish waste is quite abundant with only 50% used for human consumption, which results in the accumulation of a large amount of Ca- and HAp-rich waste (56), since about 60% to 70% of the fish bones’ weight consists of HAp [78].

So far, most of the HAp is produced by chemical synthesis. There are several methodologies to produce HAp: (1) the reaction between calcium (Ca) and phosphorus (P) salts [79], (2) synthesis through microwave radiation using calcium nitrate tetrahydrate and sodium phosphate dibasic anhydrous [78], and (3) hydrothermal and precipitation methods [80,81]. However, to mimic the mineral component of bone, and following the recent orientations of circular economy with zero waste in Agrofood chain, various biological-wastes/by-products have been used as natural sources to obtain biological-like HAp for biomedical applications usually using calcination as the main processing technique [82]. In this way, economic and environmental benefits can be retrieved through waste and/or by-products recovery [83]. Moreover, HAp prepared from natural-origin materials exhibit better biological properties due to the presence of beneficial cations (e.g., Na^+^, Zn^2+^, Mg^2+^, K^+^, and Al^3+^) or anions (e.g., F^−^, Cl^−^, SO_4_^2−^, and CO_3_^2−^) or the presence of both is proven to be even better for different biomedical applications, especially for bone regeneration [84].

#### 3.1.2. The Potential of Hydroxyapatite (HAp) for Biomedical Applications

HAp is chemically similar to the mineral component of bones in mammals, and because of that, an interesting candidate for bone reconstruction [84]. Although its main applications consist of bone repair, filler to reconstruct bone defects, or coatings for implants to promote bone ingrowth in maxillofacial, dental, and orthopedic applications, HAp can be also used as orbital implants in ophthalmology, drug delivery, percutaneous devices, and artificial blood vessels [76,77,84]. The attractive features of HAp to be used in bone-related applications include non-cytotoxicity, non-inflammatory behavior, non-immunogenic and direct bonding with new bone without requiring intermediate connective tissues [66,84].

When implanted in an osseous site, bone bioactive materials such as HAp and other CaP provide an ideal environment for cell adhesion and colonization with high osteoconductivity (support bone growth and encourage the ingrowth of surrounding bone), as well as osteoinductivity (promote the differentiation of progenitor cells to the osteoblastic lineage) [81].

HAp material presents low fracture toughness, poor tensile strength and wear resistance, as well as brittleness, which compromise its use by itself for bone regeneration [67]. One strategy is to incorporate polymers such as polylactic acid, collagen, polyethylene, and chitosan to HAp to enhance its tensile strength.

## 4. Natural-Origin Polymers

Biodegradable polymers, either synthetic or natural, are the most appropriate substrates for cell attachment, growth, and maintenance of the differentiated phenotype [61]. Natural-origin polymers have a special interest due to their biological and chemical similarities to native tissues [61]. Polysaccharides (e.g., starch, alginate, chitin/chitosan, hyaluronic acid derivatives, chondroitin sulfate, and carrageenan) and proteins (e.g., soy, collagen, fibrin, and silk) have been frequently proposed for tissue engineering applications [85]. Moreover, their resemblance with the extracellular matrix make them very attractive for tissue engineering applications, mainly because they play an important role in cell morphology, modulation, and differentiation [61]. This can be explained by a large number of cytokines/growth factors linked to glycosaminoglycans (GAGs)—extracellular matrix compound—modulating their action [85].

### 4.1. Chitosan—Sources and Extraction

Chitin, the source of chitosan, is the second most abundant natural biopolymer and the main compound of the outer skeleton of crustaceans. It is also present in the outer skeletons of insects and in the cell wall of fungi and yeast [86].

**Figure 3:**
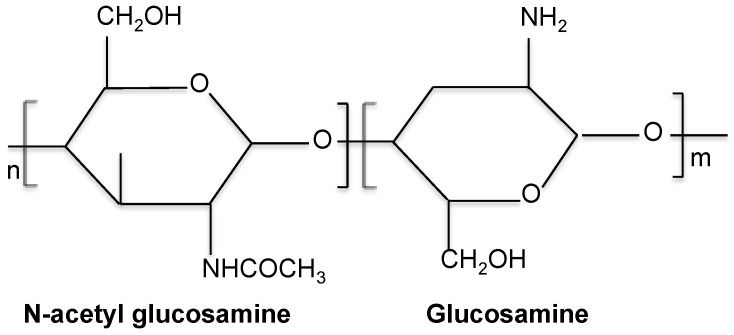
Schematic representation of chitosan chemical structure.

Chitosan is a linear hydrophilic amino polysaccharide obtained after partial alkaline deacetylation of chitin [86]. In this process, called deacetylation, some or acetyl groups are removed from the polymer (61). The degree of deacetylation (DD) is the ratio of glucosamine to N-acetylglucosamine units. When the DD of chitin reaches approximately 50% (depending on the origin of the polymer and the distribution of acetyl groups along the chains), it becomes soluble in acidic aqueous solutions and the resultant co-polymer, N-acetyl glucosamine with β (1-4) link, is designated chitosan (Figure 3). This process releases amines (NH2) which gives chitosan a cationic character [61].

Chitin and chitosan are commercially interesting polysaccharides because of the presence of the amino functionality and their high nitrogen content (6.89%) [86]. However, applications of chitin are limited compared to chitosan because it is chemically inert and insoluble in both water and acid, while chitosan is relatively reactive [87], presenting more biological properties than chitin.

#### 4.1.1. Chitosan Structure

Chitosan has three types of reactive functional groups, an amino group at C-2 and hydroxyl groups at C-3 and C-6. These groups can be tailored depending on the required properties for specific applications [87]. The amino functionality can be modified through chemical reactions such as acetylation, quaternization, reactions with aldehydes and ketones, alkylation, grafting, chelation of metals, etc., generating non-toxic and non-allergenic products with high biocompatibility and biodegradability and with several biological properties such as antimicrobial, anti-acid, anti-ulcer, anti-inflammatory, among others, [88]. In addition, the hydroxyl groups can be modified in order to increase the solubility. On the other hand, the high nitrogen content of chitosan makes it a useful chelating agent [61,88].

#### 4.1.2. Antimicrobial Potential

Chitosan has demonstrated to be efficient towards Gram-negative and Gram-positive bacteria, and for that, has been extensively used as an antimicrobial agent [89]. The polymer can penetrate the cell wall of bacteria, combine with bacterial DNA, inhibiting the synthesis of mRNA and DNA transcription [90]. High molecular weight (Mw) chitosan shows to be effective to kill bacteria due to the interaction (trough electrostatic interactions) of its cationic amino groups with the anionic negatively charged molecules (glycosaminoglycans, proteoglycans, and other negatively charged molecules) at the surface of bacteria. This mechanism leads to alteration in cell permeability and/or to create an impermeable layer around the cell, impeding the transport of essential solutes into the bacteria, promoting its death [91]. However, the antimicrobial activity of chitosan is influenced by several factors, such as microbial factors (species and cell age), environmental factors (pH, temperature, and time), physical state (soluble and solid state), and chitosan intrinsic factors (DD), positive charge density, Mw, hydrophilic/hydrophobic characteristics, and chelating capacity [92].

Regarding the microbial factors, the age of the cell can influence the antimicrobial activity of chitosan. Late-exponential phase cells of S. aureus are more vulnerable to a chitosan derivative than cells in the stationary phase and in mid-exponential phase [93]. On the other hand, cells of E. coli in the mid-exponential phase were considered most susceptible to other chitosan derivatives, followed by cells in the late-exponential phase and stationary phase [94]. The inhibitory effect of chitosan varies against different fungi, Gram-positive and Gram-negative bacteria [90]. Some authors mentioned that the effectiveness of chitosan on Gram-positive or Gram-negative bacteria is controversial [85]. Some of them stated that chitosan is more efficient for Gram-positive bacteria than for Gram-negative [89]. However, some studies revealed that the surface characteristics of the cell wall, namely hydrophilicity and charge, are closely related to the antibacterial activity of chitosan [89]. The higher hydrophilicity of Gram-negative compared to Gram-positive bacteria, makes them more susceptible to chitosan [86]. More adsorbed chitosan result in greater changes in cell wall structure and in cell membrane permeability, as aforementioned. Moreover, environmental factors also have impact: the greatest antibacterial activity of chitosan is at acidic pH values [89]. Chitosan is a cationic polymer dependent of pH, being insoluble in aqueous solutions above pH 7.5, but soluble in acidic environment (e.g., dilute and weak acids) at pH < 6 [90]. The storage temperature of chitosan solutions also affects its anti-microbial effectiveness: at 25 °C, chitosan possess the same or lower antibacterial activity when compared with solutions stored at 4 °C. Usually, fresh chitosan solutions show higher antibacterial activity compared to those stored for several weeks [95]. The physical state of chitosan also influences its activity: soluble chitosan has an extending conformation, which explains why it is more efficient at inhibiting bacterial growth; solid chitosan only contacts with the solution through the exposed surface, which leads to a lower antibacterial effect [95].

The DD influences the physical and chemical properties of chitosan such as solubility, crystallinity, swelling behavior, and biological properties, namely biodegradation by lysozyme [96], wound healing [97], osteogenic enhancement [98], and fibroblast and keratinocyte adhesion and proliferation [99]. Higher DD rises the number of free amino groups, leading to an increase in positive charge density and hence higher antimicrobial effect [100]. A higher DD of chitosan membranes corresponds to a stronger cell adhesion, allowing electrostatic interactions with the negatively charged surface of the cell membrane [100]. The DD of chitosan also affects positive charge density, conducting strong electrostatic interactions with the negatively charged bacterial surface [100].

Chitosan with different Mw possesses a different number of N-acetylglucosamines units, which influences intramolecular and intermolecular interactions [91]. Zheng and collaborators [101] showed that for Gram-negative S. aureus, the higher Mw of chitosan increases the antimicrobial activity. Other studies showed that lower Mw chitosan greatly inhibits the growth and proliferation of MO [90]. It has been also suggested that Mw has a superior effect than DD on the antimicrobial activity [102].

Chitosan has a high chelating capacity for metal ions (including Ni^2+^, Zn^2+^, Co^2+^, Fe^2+^, Mg^2+^, and Cu^2+^), where the amino groups are responsible for the uptake of metal cations [92]. In this sense, chitosan selectively binds to trace metals and thereby inhibits the production of toxins and microbial growth [101]. This mechanism is more efficient at higher pH, where positive ions are bonded to chitosan and the amino groups are not protonated, releasing the electron pair on the amine nitrogen to bind to metal ions [95].

#### 4.1.3. Biodegradation

Chitosan’s biodegradability in vivo depends on several factors, such as Mw and DD [61]. Typically, the chitosan degradation rate increases as the DD decreases [61]. Chitosan is degraded by specific enzymes that hydrolyze linkages between glucosamine–glucosamine, glucosamine–N-acetyl-glucosamine, and N-acetyl-glucosamine–N-acetyl-glucosamine units [61]. Chitosan polymer is hydrolyzed by specific enzymes (e.g., chitosanases). These enzymes exist in MO [101], but are absent in mammals. Chitosanases catalyze the endohydrolysis of ß-1,4-linkages between D-glucosamine residues in chitosan molecules [103]. In humans, chitosan degradation occurs mostly by lysozyme and bacterial enzymes present in the colon. Lysozyme is quite ubiquitous in the human body and an important effector in the inflammatory response, secreted by several inflammatory cells, such as macrophages, monocytes, and granulocytes [104]. After oral administration, chitosan degrades in the gastrointestinal tract [105].

#### 4.1.4. Potential of Chitosan for Biomedical Applications

The cationic nature of chitosan confers to this polymer unique properties with a widespread range of applications namely in food, agriculture, water and waste treatment, and cosmetic industries [86,88]. Furthermore, it is a potential biomaterial for biomedical applications due to its biocompatibility (minimizes additional local inflammation) [105], biodegradability [9,100], wound healing ability [100], and hemostatic properties [106]. Chitosan shows low toxicity with versatile biological activities such as antimicrobial activity [94], low immunogenicity [107], anti-thrombogenic agent [103]. Chitosan can be used either alone or in combination with other natural-origin polymers [108], such as aliphatic polyesters [109,110] and with ceramics such as HAp [111,112].

Due to its cationic nature and predictable degradation rate, chitosan-based materials can bind growth factors and release them in a controlled manner, which is suitable for bone tissue engineering [61]. Chitosan has shown to support the differentiation, proliferation, and mineral rich matrix deposition by bone marrow stromal cells in culture [109]. Chitosan is easily processed into gels [113], membranes [114,115], nanofibers [116], microparticles [117], nanoparticles [118], and scaffolds [109] for several applications.

The solubility of chitosan depends on its biological origin, pH, distribution of free amino and N-acetyl groups along the chain, molecular weight, as well as the DD [87]. As the DD decreases, the degree of solubility in solvents is lower [88]. Pure native chitosan (pKa~6.3) is insoluble in water, alkaline medium, and common organic solvents [61]. Nonetheless, it dissolves in aqueous inorganic and acidic solutions below its pKa (~6.3) (e.g., acetic acid, formic acid, succinic acid, lactic acid, and malic acid) [119]. Regarding the solution viscosity, this is affected by the DD, Mw, concentration, the ionic strength of the solvent, cationic character, pH, and temperature [61]. Generally, an increase in temperature causes a decrease in the viscosity of the solution. The higher Mw chitosan often render highly viscous solutions [87]. Commercially, chitosan is available with >85% deacetylated units (degree of acetylation < 15%), and Mw between 100 and 1000 kDa [93].

## 5. Chitosan-Hydroxyapatite Biomaterials

The functional groups of chitosan allow it to interact with various materials, such as HAp, forming composites for bone regeneration. The cationic nature of chitosan constitutes the foundation of its potential applications, as a linear polyelectrolyte with a high charge density that can interact with negatively charged molecules, like proteins.

Chitosan-hydroxyapatite composite presents interesting mechanical properties due to the attachment of amino and hydroxyl groups of chitosan to the calcium ions present at the surface of the HAp crystals [119].

In literature, there are many reports about conjugations of chitosan and hydroxyapatite in numerous forms. Table 3 summarizes works describing chitosan and hydroxyapatite formulations such as pastes, coatings, particles, scaffolds, and hydrogels for orthopedic and tissue engineering applications.

Chitosan-HAp composites can be produced in several forms: pastes [120,121,122,123,124,125], coatings [126,127,128,129,130], particles [111,123,131,132], scaffolds [133,134,135], hydrogels [122], and films [136,137]. The methods employed to produce chitosan-HAp composites for orthopedic and tissue engineering applications are considerable and depend on the type of structure. The simple mixture of HAp into a chitosan solution drives to the formation of a paste [120,121,122,124,125] that can be used for bone regeneration or to carry osteogenic inducing factors, such as bone marrow aspirate, BMP-2, or cells [124]. Chitosan-HAp coatings are mainly produced to be deposited over a titanium or other metallic substrate (simulating prosthetic materials) by spraying [126], electrophoretic deposition [127], electrochemical deposition [128,129], or sol-gel process [130]. Chitosan-HAp particles can be microparticles produced by spray-drying achieved [111,132] or nanoparticles generated by precipitation [123] or in situ hybridization [131]. Composite scaffolds, hydrogels, and films/membranes are considered 3D structures, and numerous fabrication methods are described in the literature. Freeze drying is one of the most utilized to produce scaffolds [133], although other techniques can be used, such as using enzymes to degrade chitosan [134] or by textile methodology NSN to produce fibrous structures [135]. Membranes or films are easily achieved by producing a hydrogel [112], which is further dried [136,137].

Joint replacement demands a structure with high mechanical properties, which is not the case of any of the described forms of chitosan-HAp composite. For the purpose of preventing PJI, a chitosan-HAp composite in the form of a coating or paste to cover the metallic implant would be the most appropriate. This composite could be used as a bioactive interface between the implant and patient’s bone: chitosan to prevent the development of PJI; and HAp by enhancing osteoconduction and osteoinduction of the implant, boosting formation of new bone.

## 6. Conclusions and Future Perspectives

Periprosthetic joint infection is one of the most serious complications after orthopedic surgery and a critical mechanism of failure, which leads to complex revision procedures. Only a small number of MO are needed to infect the implant, resulting in the formation of a microbial biofilm on the surface. Standard treatments include the removal of the implant, but also the removal of necrotic bone pieces, local and/or systemic administration of antibiotics, and also a revision arthroplasty with a new prosthesis, when the infection is eradicated. Therefore, an efficient alternative to the conventional therapeutics would be the incorporation of natural antimicrobial compounds into the prosthetic materials.

Natural-origin material such as chitosan is a suitable option for this application mainly due to its intrinsically antimicrobial properties to a broad antimicrobial activity including Gram-negative and Gram-positive bacteria and fungi. The combination of this material with osteoregenerative HAp to develop sustainable solutions for PJI prevention and treatment, without recurring to the conventional antibiotic therapies, is considered an opportunity. Although the majority of CaP-based materials are synthetic, which leads to increased consumption of phosphate depleting many of the natural resources of phosphorous in a near future, HAp can be extracted from natural sources, such as fish bones or scales, which are by-products of the fish food industry, promoting the circular economy guidelines.

Solutions in regenerative medicine, namely in the orthopedic field either for bone regeneration, or for the treatment of bone associated infections, should rely on natural sustainable materials to improve our world with greener and recyclable related technologies and avoid extinguishing natural sources.

## Figures and Tables

**Table 1:** Types of periprosthetic joint infections.

Types of PJI	Development	Causes	Treatment
**Early/acute**	≤3 months	Usually start at the time of surgery through intra-operative contamination.	Attempt at debridement and prosthetic retention
**Delayed/subacute**	3–24 months	Can also be acquired at the time of surgery, however caused by less virulent MO.	Attempt at debridement and prosthetic retention or removal
**Late/chronic**	≥24 months	Can be initially asymptomatic, but also be caused at the time of surgery. Frequently caused by hematogenous infection.	Prosthetic removal

**Table 2:** CaP-based compounds, Ca/P ratio, and pKs values [66,67].

Compound	Ca/P Ratio	Application	Reference
Monocalcium phosphate monohydrate	0.5	Increase root fluoride uptake	[68]
Monocalcium phosphate anhydrous	0.5	Artificial bone graft	[69]
Dicalcium phosphate anhydrous	1	Polish agent for teeth Source of Ca and P in food supplements	[70]
Dicalcium phosphate dihydrate	1	Sustained release of highly water-soluble drugs	[71]
α-Tricalcium phosphate	1.5	Biodegradable composite for bone repair	[72]
β-Tricalcium phosphate	1.5	Orthopedic surgery	[73]
Calcium-deficient hydroxyapatite	1.5–1.6	Bone grafting	[74]
Hydroxyapatite	1.67	Repairing of hard tissues	[75]
Fluorapatite	1.67	Used as source of fluorine in pharmaceutical products	[76]
Tetracalcium phosphate	2	Applied as cements and coatings on metallic implants	[77]

**Table 3:** Studies showing the combination of chitosan and hydroxyapatite-based materials for orthopedic and tissue engineering applications.

Material	Year	Material Preparation	Methodology	Main Achievements	Reference
HAp, ZnO, CaO—Ch paste	1991	HAp, ZnO, and CaO powders in different percentages mixed with Ch solution to form a paste.	Characterization of the paste: pH, setting time, compressive strength, morphological observation, and X-ray diffraction analysis.	The paste with 92% of HAp, 6% of ZnO, and 2% of Cao evidenced the best results: neutral pH, short setting time, and relatively high compressive strength and slightly elastic consistency.	[120]
HAp, ZnO, CaO —Ch paste	1992	HAp, ZnO, and CaO powders mixed with Ch solution to form a paste.	Bone defect in rabbits. Histological and X-ray follow up until 20 weeks.	Radiographic examination revealed that a bone-like irregular radiopacity appeared in the region of the embedded paste. This was judged histopathologically as the formation of bone tissue with chondral tissue.	[121]
Ch-bonded self-hardening paste with HAp granules	1996	Ch-HAp hardened composite paste was compared with tibial cancellous bone and PMMA bone cement.	Characterization of the paste and comparison with human cancellous bone and PMMA bone cement: pH, exothermic temperature profile, and compressive strength.	Kneading and setting of the paste generated a little amount of heat (32.8 °C) as compared with the heat produced by bone cement (114.5 °C). The pH value of Ch-HAp was nearly equal to human plasma. The strength was comparable to that of the cancellous bone, but lower than bone cement.	[122]
Ch-HAp nanocomposites	2002	The Ch-HAp nano-composite was prepared by precipitation method.	Characterization of composites crystal phases: X-ray diffractometer and infrared spectrophotometry. The HAp particles were observed by electron microscopy and their specific surface area by BET.	Nano-sized HAp/Ch nano-composites presented a homogeneous microstructure.	[123]
Ch glutamate and HAp paste	2003	HAp and Ch glutamate at a ratio of 4:1 were used to make the paste as a delivery vehicle for autologous BM aspirate, BMP-2, and osteoblasts grown from the autologous BM aspirate.	Rat calvaria critical size defect. New bone formation was analyzed by histology. BMD and mechanical properties were also assessed.	HAp and Ch glutamate paste containing osteoblasts cultured from BM aspirate presented the best results.	[124]
Ch-nHAp composite scaffold	2005	Freeze-drying	Characterization of the scaffold micro-structure and physical and chemical properties were studied by using SEM, porosity measurement, TGA, XRD, XPS, and FTIR. In vitro biocompatibility assessment with MC3T3-E1 cells.	The spongy scaffolds showed good porosity and some cells could grow in the pores.	[133]
Porous Ch-HAp hybrid scaffold	2006	Developed by partial enzymatic degradation of the Ch surface using chitosanase and lys.	Characterization of the scaffold by SEM and biological interaction with L929 cells.	The presence of HAp and porosity produced by partial lys hydrolysis enhanced cell proliferation. Besides, cell adhesion and proliferation are primarily dependent on substrate roughness and stability.	[134]
Ch–HAp hydrogel composite membranes	2009	HAp was deposited on the surface of Ch hydrogel membranes by a wet chemical synthesis method by alternatively soaking the membranes in CaCl_2_ and Na_2_HPO_4_ solutions for different time intervals.	The surface deposition of HAp was analyzed. Biological interaction with MG-63 cells was evaluated.	HAp deposition occurred on the surface of Ch hydrogel membranes within a short period of time (20 h). Biocompatibility studies results showed excellent cell viability.	[136]
Ch-HAp multi-layered film	2010	Sublimation/compression method, free-standing nacre-like composite films were prepared with polymeric repulsion control of an organic/inorganic solution.	Characterization of the films using SEM, TGA, XRD, FTIR and by assessing the mechanical properties.	A HAp to Ch ratio of 100 w/w% was found to be optimal for preparation of the highest flexible film.	[137]
Antibacterial Ch-HAp complex coatings	2011	Porous HAp coatings prepared by liquid precursor plasma spraying process were used for loading n-carboxymethyl Ch with different concentrations.	Antimicrobial assessment with S. aureus and cytotoxicity evaluation with MG-63 cells.	The antibacterial efficacy on *S. aureus* increases proportionately with Ch concentration. However, coating with low Ch concentrations (10 and 20 g/L) also exhibited enhanced proliferation of osteoblast cells, indicating a concentration window for selective destruction of bacteria.	[126]
nHAp and Ch-HAp nanocomposites	2013	Nano-HAp composites with different Ch content were prepared via in situ hybridization route.	Characterization of the composites by SEM, AFM, FTIR, water contact angle, mechanical testing, and in vitro bioactivity analyses. In vitro evaluation of cell viability and osteogenic differentiation using USSC. In vivo bone regeneration using the rat calvarial defect.	nHAp powder with lower surface roughness, higher surface wettability, and more narrow size distribution and smaller particle size than composite powders and next to it, nHAp/Ch could adsorb more protein and hence more osteogenic signal expression and bone regeneration ability than other nanocomposite powders in vitro and rat, respectively.	[131]
Ch-carbonated HAp composite coatings	2014	Preparation of CCCs on Ti6Al4V substrates by electrophoretic deposition; transformation of CCCs into CHACs in PBS; formation of CCHCs by modification of CHACs with Ch.	Characterization of the coatings by SEM, TGA, XRD, FTIR, water contact angle. In vitro biocompatibility assessment using hBMSCs.	The hBMSCs show better cell morphology, adhesion, spreading, and proliferation on CCHCs than on CHACs. The excellent biocompatibility of CCHCs is mainly attributed to the organic/inorganic compositions, macroporous structure, and moderately hydrophilic surfaces.	[127]
Ch-HAp biocomposite microspheres	2015	Ch-HAp microspheres were prepared by co-precipitation method using a spray-dryer as a drying medium.	Characterization of the microspheres by SEM, XRD, FTIR, surface area by BET, and bioactivity studies.	Spray-dried mesoporous microspheres with high surface area were successfully produced. Nucleation and growth of apatite are enhanced using Ch.	[132]
Ch-nHAp reinforced composite hydrogel	2015	Injectable thermosensitive hydrogel containing Zn-Ch/nHAp/β-GP produced by sol-gel method.	Characterization of the hydrogels by SEM, EDS, XRD, FTIR, protein adsorption, and bioactivity tests. In vitro biocompatibility and osteogenic assessment using mBMSCs. In vivo rat critical-sized tibial defect model.	The hydrogel exhibited sol–gel transition at 37 °C. It was non-toxic to cells and osteoconductive, promoting the differentiation of mMSCs into osteoblasts. Zn-Ch/nHAp/β-GP hydrogels promoted bone healing in critical-sized rat tibial defects.	[112]
Ch-nHAp hybrid microparticles	2016	Nanodispersions of nHAp in the presence of Ch were produced) with subsequent spray-drying of microparticles.	Particle size characterization of obtained dispersions. Characterization of microparticles by SEM, EDS, XRD, TGA, and DSCA.	Production of homogeneous and stable nanodispersions, and the subsequent spray-dried microparticles, incorporating highly pure HAp nanoparticles of approximately 50 nm, without degrading chitosan.	[111]
Ch-HAp paste	2017	Ch was mixed with artificial HAp and dried for 24 h. Then, saline physiologic solution was added to the Ch-HAp to obtain a paste.	The humidified Ch-HAp paste was applied to the oral bone defect in human patients. A small sample was removed after 3 months. Histological analysis, X-ray, and µCT was performed to evaluate the bone regeneration.	The Ch-HAp implant reduced the pocket depth of the supporting tissue. It also reduced the grading of tooth mobility and promoted alveolar bone growth.	[125]
Ch-HAp-Ag coatings	2017	*In situ* codeposition of HAp-NPs and Ag-NPs on Ti surface with Ch, driven by pulse electrochemistry, to obtain a coating on a Ti matrix surface.	Friction properties and ion release tests, and bioactivity studies of the coatings. Antibacterial testing with *E. coli* and *S. aureus.*	Composite coating with Ch-mediated HAp and Ag-NPs exhibited good antiwear properties and long-term antibacterial performance.	[128]
Lys/Ch/Ag/HAp hybrid coatings	2018	Lys/Ch/Ag/HAp hybrid coating was successfully fabricated on Ti surface by electrochemical deposition method and spin coating process.	Characterization of the coatings by SEM, TEM, EDS, XRD, and XPS. Antibacterial testing with *E. coli* and *S. aureus.* Viability/osteogenic assessment using MC3T3-E1 cells.	Coatings presented a hierarchical nanostructure with a uniform distribution of Lys, Ch, Ag, and HAp. They also presented in vitro antibacterial and no cytotoxicity.	[129]
Ch fiber scaffold functionalized with organically modified ormoHAp	2019	Ch fibers produced by NSN technique were functionalized in two ways: collagen type I coating and ormoHAp.	Biocompatibility and osteogenic assessment using hBMSCs.	NSN scaffold functionalization with collagen and ormoHAp improved attachment, proliferation, and differentiation of hBMSC.	[135]
Ch-HAp nanocomposite coatings	2020	Ch-HAp nanocomposite coatings with increasing concentrations of HAp were deposited through sol-gel process on alkali-treated Ti6Al4V substrate.	Characterization of the coatings by SEM, XRD, FTIR, water contact angle and adhesion strength measurements, and bioactivity studies. Proliferaton studies with hBMSCs.	Increasing HAp content led to a higher surface roughness. Bioactivity of the Ch/HAp nanocomposite coatings enhanced bone-like apatite layer formation on the material surface with increasing HA content. Ch/HAp nanocomposite coatings were biocompatible, in particular the Ch/10 wt.% HAp composition.	[130]

Ch, chitosan; HAp, hydroxyapatite; nHAp, nano hydroxyapatite; Ag, silver; β-GP, beta-glycerophosphate; ormoHAp, organically modified hydroxyapatite ZnO, zinc oxide; CaO, calcium oxide; NaOH, sodium hydroxide; SEM, scanning electron microscopy; TEM, transmission electron microscope; TGA, thermogravimetric analysis; XRD, X-ray diffraction; XPS, X-ray photoelectron spectroscopy; FTIR, Fourier transformed infrared spectroscopy; EDS, energy dispersive spectroscopy; AFM, atomic force microscope; DSC, differential scanning calorimetry; NPs, nanoparticles; Ti, titanium; BMD, bone mineral density; BET, Brunauer, Emmett, and Telleru method; µCT, micro computed tomography; CCHCs, chitosan/carbonated hydroxyapatite composite coatings; CCCs, calcium carbonate coatings; CHACs, carbonated hydroxyapatite coatings; NSN, net-shape-nonwoven; 3D, tridimensional; LDH, lactate dehydrogenase; MC3T3-E1, mouse C57BL/6 calvaria cell line; L929, mouse fibroblast cell line; SFB, simulated body fluid; m-SBF, modified simulated body fluid; CHO, Chinese hamster ovary cell line; TE, tissue engineering; HEPM, human palatal mesenchyme cell line; SAOS-2, human osteosarcoma cell line, MG-63, human osteosarcoma cell line; Lys, lysozyme; BMP-2, bone morphogenetic protein 2; BM, bone marrow; hBMSCs, human bone marrow mesenchymal stem cells, mMSCs, murine mesenchymal stem cells; USSC, umbilical cord stem cells; *E. coli*, *Escherichia coli*; *S. aureus*, *Staphylococcus aureus*.

## Data Availability

Data sharing not applicable. No new data were created or analyzed in this study. Data sharing is not applicable to this article.
